# Telephone based self-management support by ‘lay health workers’ and ‘peer support workers’ to prevent and manage vascular diseases: a systematic review and meta-analysis

**DOI:** 10.1186/1472-6963-13-533

**Published:** 2013-12-27

**Authors:** Nicola Small, Christian Blickem, Tom Blakeman, Maria Panagioti, Carolyn A Chew-Graham, Peter Bower

**Affiliations:** 1 Greater Manchester Collaboration for Leadership in Applied Health Research and Care, Centre for Primary Care, and Manchester Academic Health Science Centre, University of Manchester, Manchester, UK; 2 Research Institute Primary Care and Health Sciences, Keele University, Keele, UK

**Keywords:** Self-management support, Chronic care, Non-healthcare professional, Peer, Lay, Telephone, Vascular disease, Chronic kidney disease, Prevention, Management, Patients, Social disadvantage, Health services research, Randomised controlled trial, Review, Meta-analysis

## Abstract

**Background:**

Improved prevention and management of vascular disease is a global priority. Non-health care professionals (such as, ‘lay health workers’ and ‘peer support workers’) are increasingly being used to offer telephone support alongside that offered by conventional services, to reach disadvantaged populations and to provide more efficient delivery of care. However, questions remain over the impact of such interventions, particularly on a wider range of vascular related conditions (such as, chronic kidney disease), and it is unclear how different types of telephone support impact on outcome. This study assessed the evidence on the effectiveness and cost-effectiveness of telephone self-management interventions led by ‘lay health workers’ and ‘peer support workers’ for patients with vascular disease and long-term conditions associated with vascular disease.

**Methods:**

Systematic review of randomised controlled trials. Three electronic databases were searched. Two authors independently extracted data according to the Cochrane risk of bias tool. Random effects meta-analysis was used to pool outcome measures.

**Results:**

Ten studies were included, primarily based in community settings in the United States; with participants who had diabetes; and used ‘peer support workers’ that shared characteristics with patients. The included studies were generally rated at risk of bias, as many methodological criteria were rated as ‘unclear’ because of a lack of information.

Overall, peer telephone support was associated with small but significant improvements in self-management behaviour (SMD = 0.19, 95% CI 0.05 to 0.33, I^2^ = 20.4%) and significant reductions in HbA1c level (SMD = -0.26, 95% CI −0.41 to −0.11, I^2^ = 47.6%). There was no significant effect on mental health quality of life (SMD = 0.03, 95% CI −0.12 to 0.18, I^2^ = 0%). Data on health care utilisation were very limited and no studies reported cost effectiveness analyses.

**Conclusions:**

Positive effects were found for telephone self-management interventions via ‘lay workers’ and ‘peer support workers’ for patients on diabetes control and self-management outcomes, but the overall evidence base was limited in scope and quality. Well designed trials assessing non-healthcare professional delivered telephone support for the prevention and management of vascular disease are needed to identify the content of effective components on health outcomes, and to assess cost effectiveness, to determine if such interventions are potentially useful alternatives to professionally delivered care.

## Background

Improving prevention and management of vascular disease is a global priority [1], particularly in areas of socio-economic disadvantage where cardiovascular disease accounts for around 33% of the gap in life expectancy [2]. Models of service delivery, such as the Chronic Care Model [3], advocate a whole systems approach to improve quality of care. Recommended strategies include addressing cardiovascular risk [2,4], and the provision of effective self-management support.

There is evidence to suggest that there are limits to the ability of health care professionals to provide effective self-management support, especially for patients who are socially disadvantaged [5]. Non-health care professionals are increasingly being used alongside conventional services to offer additional support, to reach disadvantaged populations and to provide more efficient delivery of care [6,7].

We distinguish two, related groups of non-health care professionals - ‘lay health workers’ and ‘peer support workers’. Lewin defines a ‘lay health worker’ as:

*‘… usually provided with job-related training, they have no formal professional or paraprofessional tertiary education, and can be involved in either paid or voluntary care. The term ‘LHW’ is thus necessarily broad in scope and includes for example, community health workers, village health workers, cancer supporters and birth attendants’*[8].

A subset of lay health workers are ‘peer support workers’ which Dale defines as:

*‘… a source of support, internal to a community, who share salient target population similarities (e.g. age, ethnicity, health concern, or stressor) and possess specific knowledge that is concrete, pragmatic and derived from personal experience rather than formal training’*[9].

Delivering effective self-management support can be problematic in patient populations with poor access to healthcare because of geographical location, mobility issues, or competing demands on their time. More effective use of the telephone and other communication devices could provide a more effective way of providing care. A number of randomised controlled trials (RCTs) assessing the efficacy of structured telephone support currently exist, and patients with diabetes and heart failure are the populations most frequently researched [10-12].

Two Cochrane reviews have assessed the efficacy of structured telephone support for different long-term conditions [13,14]. The first review assessed 7 interventions delivering telephone support by ‘peer support workers’ to patients with different types of acute and long-term conditions, and reported improvements in behavioural outcomes, such as mammography (screening for breast cancer) and changes in diet [13]. However, there were no significant differences between peer support interventions and usual care groups in self-efficacy, health status and mental health outcomes [13].

The second Cochrane review assessed 25 studies focused on the efficacy of structured telephone support and telemedicine delivered by healthcare professionals to patients with chronic heart failure [14]. Both interventions reduced hospitalisations, and several studies improved patient outcomes (such as quality of life) and reduced healthcare costs [14].

Although these studies have demonstrated improvements in outcomes, significant questions remain as to the impact on the prevention and management of vascular disease, the frequency and dose of contacts most likely to be effective, and the relative impact of different types of telephone support, such as emotional, appraisal and informational support [15].

The authors are currently involved in the development and evaluation of a ‘lay health worker’ led telephone self-management support intervention, which is being evaluated in an RCT in people diagnosed with stage 3 chronic kidney disease (CKD [16]). The trial is being carried out as part of the NIHR Collaboration for Leadership in Applied Health Research and Care (CLAHRC) for Greater Manchester, which aims ‘to improve healthcare and reduce inequalities in health for people with chronic vascular conditions (diabetes, heart disease, kidney disease and stroke’: http://clahrc-gm.nihr.ac.uk/).

Improving the delivery of care for people with CKD is essential to reducing cardiovascular morbidity and mortality [17]. CKD is a relatively recent disease classification and comprises 5 main stages [4,18]. Alongside other long-term conditions, such as diabetes and hypertension, general practices in the United Kingdom (UK) are incentivised to establish registers and provide evidence-based care for patients with CKD. The early stages of CKD represent mild and moderate problems with kidney function, associated with an increased risk of progression to established kidney failure (i.e. stage 5). More commonly however, early stage CKD is associated with an increased risk of cardiovascular events. With recognition that CKD is an independent risk factor, guidelines and quality standards emphasise the need to address risk in individuals with CKD and provide relevant education and support with lifestyle change [4]. CKD is common and tends to be associated with other conditions including hypertension, diabetes and ischaemic heart disease. Therefore, identifying CKD and supporting self-management is of relevance in both primary and secondary prevention of cardiovascular disease (CVD).

The review reported here aims to synthesise published evidence on the effectiveness of non-healthcare professional (‘lay health workers’ and ‘peer support workers’) telephone self-management interventions, to inform the delivery of the BRIGHT trial as part of a modelling phase of the complex interventions development process [19]. We assumed the CKD specific literature would be limited, and based our study on the assumption that there were sufficient commonalities in the management of vascular diseases and other long-term conditions associated with vascular disease to make the results potentially generalisable across this cluster of disorders [7,20]. This study used a systematic review and meta-analysis to assess the evidence on the effectiveness and cost-effectiveness of telephone self-management interventions led by ‘lay health workers’ and ‘peer support workers’ to support the prevention and management of vascular disease.

## Methods

We followed established guidelines for conducting and reporting systematic reviews [21,22].

### Literature search strategy

The search strategy was restricted to electronic databases. We developed relevant search strings based on published work in this area [13,14].

Initially the search process involved identifying relevant published reviews, including those identified from the Cochrane Database of Systematic Reviews (CDSR) and the Database of Abstracts of Reviews of Effects (DARE).

We then used a simplified RCT search strategy which has demonstrated good performance in identifying RCTs for systematic reviews of health care interventions [23]. The strategy consists of a search of the Cochrane Central Register of Controlled Trials (CENTRAL) database, with supplementary searches of EMBASE and MEDLINE. The entire search strategy is available in Additional file 1.

There were no resources for translation and the study was restricted to English language reports.

### Inclusion and exclusion criteria

Inclusion criteria were:

1. RCTs;

2. Adults (aged ≥ 18 years) with a diagnosis of vascular disease or long-term conditions associated with vascular disease, including: cerebrovascular disease, peripheral vascular disease, ischemic heart disease, stroke, heart failure, CKD, diabetes, and hypertension. Studies dealing with subcategories of vascular disease (e.g. Buerger’s disease and blood clotting disorders), which are not currently embedded within the routine management of vascular conditions, were excluded;

3. Based on non-healthcare professional delivered telephone based self-management support.

We included two types of ‘non-health care professional’. We defined ‘lay health workers’ as an individual ‘provided with job-related training, with no formal professional or paraprofessional tertiary education’ [8]. We further distinguished the subset specifically using a ‘peer support worker’ defined as someone who ‘shares salient target population similarities (e.g. age, ethnicity, health concern, or stressor) and possesses specific knowledge that is concrete, pragmatic and derived from personal experience rather than formal training’ [24].

We included interventions delivering ‘structured telephone support’, based on verbal communication through standard telephone equipment [13]. We included ‘self-management support’ of any intensity or duration, but only when planned as part of a treatment protocol and not delivered on an ad-hoc basis. ‘Self-management’ was defined as ‘the care taken by individuals towards their own health and well being: it comprises the actions they take to lead a healthy lifestyle; to meet their social, emotional and psychological needs; to care for their long-term condition; and to prevent further illness or accidents’ [25].

Self-management support by telephone is often delivered as part of a more complex package of care. As the review was intended to assess the benefits of telephone support, we restricted inclusion to studies where self-management support by telephone was *primary* (i.e. the telephone support represented the majority of the intervention in terms of time and resources) and *distinct* (i.e. the design of the trial was such that the effects of telephone support could be distinguished).

The primary comparison was with usual or routine care for patients with vascular disease or long-term conditions associated with vascular disease. Studies comparing different types of telephone support were included as a secondary comparison.

Exclusion criteria were:

1. If the intervention was delivered by a qualified or trainee health care professional;

2. If the calls were not supportive in content (i.e. reminder calls to assess medication compliance, involving one or two questions only);

3. If the telephone support was patient initiated only (i.e. patients called the support service). For inclusion, studies had to involve program initiated calls, but could include patient initiated calls alongside program initiated ones;

4. If the intervention was home telemedicine (i.e. where the use of information technologies allow face-to-face contact through videoconferencing and may include the storage of clinical digital samples which are sent to the provider via electronic transmission such as email or via a telemedicine hub);

5. Non-English language publications.

All titles were initially screened for possible inclusion by one reviewer (NS). All abstracts passing this initial screen were examined independently by two reviewers (NS and PB) and any disagreements were resolved through discussion.

### Data abstraction

All data extraction was conducted by two members of the research team (NS and CB) working independently, with disagreements resolved via discussion.

We extracted the following data:

1. Setting: year of study, geographical and other context;

2. Participants: vascular diagnosis; other long-term conditions associated with vascular disease;

3. Intervention: relevant components of a telephone intervention (including recruitment and training of workers); content of control or comparison group;

4. Outcomes: self-management (such as, self-reported health behaviours, self-efficacy, empowerment, using validated patient reported outcome measures); generic quality of life; clinical outcomes (such as, reported blood pressure, HbA1c, and mortality); health care utilisation (such as, hospital visits and admissions, primary care visits, medication use, other health care use); cost-effectiveness.

### Study quality

Two members of the research team (NS and MP) independently extracted data according to the Cochrane risk of bias tool [22]. Thus, the following five domains were considered:

1. Sequence generation: was the allocation sequence adequately generated?

2. Allocation concealment: was the allocation adequately concealed?

3. Blinding of participants, personnel and outcome assessors for each main outcome or class of outcomes: was knowledge of the allocated treatment adequately prevented during the study? We assessed blinding of outcome assessments separately for patient reported measures, observer measures, and measures of health care utilisation.

4. Incomplete outcome data for each main outcome or class of outcomes: were incomplete outcome data adequately addressed?

5. Selective outcome reporting: are reports of the study free of suggestion of selective outcome reporting? (Based on the existence of study protocols).

A judgment was made for each domain into one of three categories – ‘low’, ‘unclear’ or ‘high’ risk of bias [22]. We tabulated quality assessments alongside other details on the included studies as above, to assess overall quality of the literature and any relationship between quality assessment and outcomes.

### Analysis

Data on the effect of structured telephone interventions on outcomes were extracted. We extracted data on the following five outcome categories: ‘self management’; ‘mental health’; ‘clinical (surrogate outcomes)’; ‘health care utilisation’ and ‘cost effectiveness’. As outcome measures varied in type (i.e. different types of self-management) or presentation (continuous HbA1c scores and dichotomous measures of proportions achieving a certain reduction), we calculated the *standardised mean difference* (SMD). We translated measures using dichotomous data to a SMD using conventional methods [26], and pooled analyses using random effects models where we judged there were more than 2 studies of sufficient similarity to make the results interpretable. We reported the I^2^ statistic to assess heterogeneity. Where there were insufficient studies to pool, we reported individual study SMDs to facilitate comparison.

Data used in the review were scores of patients who were followed-up. Cluster trials were analysed reducing effective sample size through calculation of the ‘design effect’ [22]. Each trial contributed a single estimate to each outcome category, with decisions about inclusion of multiple outcomes based on maximising comparability between studies.

## Results

**Figure 1:**
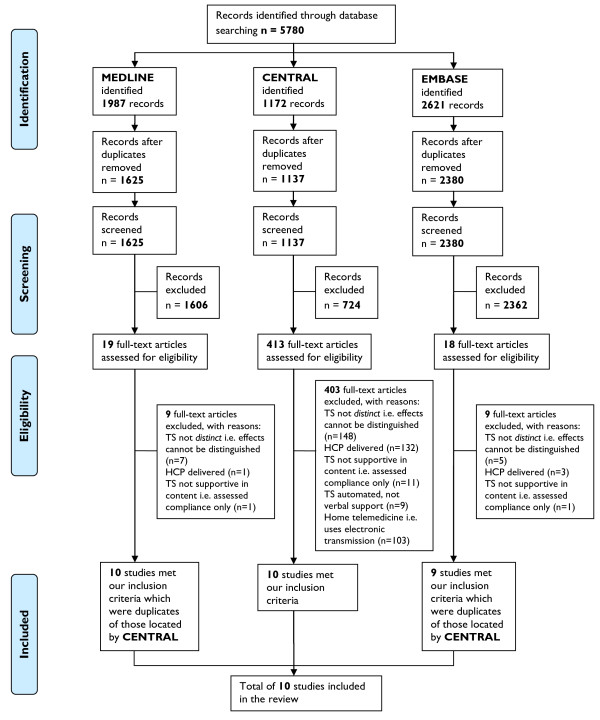
PRISMA flowchart.

Figure 1 presents the PRISMA flow chart outlining the process of study selection. Screening of 5780 titles and abstracts left 450 articles for review of full texts. In total, 10 studies were found to meet all inclusion criteria.

### Description of included studies

Table 1 shows the characteristics of the 10 included studies in the review (see Additional file 2: Table S1 for further details of the full extraction).

**Table 1:** Characteristics of included studies

**Study**	**Setting**	**Randomisation**	**Participants**	**Interventions**	**Outcomes**
**Turner 2012**[27]	US	RCT 2 arm: Patient level	280 Patients with uncontrolled hypertension based on average of measurements from visits over 2-year period; prescribed 2+ antihypertensive medication	*Peer training:* An experienced ‘lead peer coach’ demonstrated telephone support skills and techniques and 11 peers practiced calls.	SM, CO
				*Frequency of calls:* 3 months of calls by peers; on alternate months, 2 practice visits to review a personalised 4-year heart disease risk calculator and view slide shows; peers shared concerns over challenging cases.	
				*Content of calls:* Assessed patient attitudes whist giving evidence-based advice; offered role modelling and perceived behavioural control advice (informational, appraisal and emotional support).	
				*Control:* Usual care plus heart disease brochures (AHA).	
**Walker 2011**[28]	US	RCT 2 arm: Patient level	526 Patients with type 2 diabetes with HbA1c level ≥7.5%; prescribed one or more oral medications.	*Training:* ‘Non clinical health educators’ were trained by a diabetes educator nurse.	SM, PROMS
				*Frequency of calls:* 10 calls at 4–6 week intervals over 12 months.	
				*Content of calls:* Callers used a manual developed to improve self-efficacy and empowerment (informational, appraisal and emotional support). Peers encouraged patients to choose from topics including diabetes medication adherence and addressing and maintaining lifestyle changes through healthy eating and physical activity.	
				Calls were tailored to each patient.	
				*Control:* Received high quality self-management materials by mail.	
**Heisler 2010**[29]	US	RCT 2 arm: Patient level	244 Patients with type 2 diabetes with HbA1c level ≥7.5% during previous 6 months	*Peer training:* 125 peers attended a group session to set diabetes goals, receive peer communication skills training, and receive support from an age-matched ‘peer partner’.	SM, PROMS, HU
				*Frequency of calls:* Peers were encouraged to talk weekly using a telephone that recorded call occurrence. Optional patient-led group sessions at 1, 3, and 6 months.	
				*Content of calls:* Sharing education; emotional concerns and progress on self-management; and motivational interviewing (informational, appraisal and emotional support).	
				*Control:* Enhanced usual care consisting of an educational session plus support via nurse care manager.	
**Dale 2009**[9]	UK	*RCT 3 arm: Patient and nurse level	231 Patients with type 2 diabetes with inadequate glycaemic control (raised HbA1c level).	*Peer training:* 9 ‘Peer supporters’ and 12 practice nurses attended a communication skills training programme and delivered calls for 6 months.	SM, PROMS, HU
				*Frequency of calls:* The first call was made 3–5 days later and the following days: 7–10, 14–18, 28–35, 56–70, 120–50.	
				*Content of calls:* Sharing education; increasing self-efficacy, empowerment, and self-management; facilitating goal-setting and active listening, including motivational interviewing (informational, appraisal and emotional support).	
				*Control:* Usual care.	
**Samuel-Hodge 2009**[30]	US	RCT 2 arm: Cluster by 24 churches	201 Patients with type 2 diabetes, defined as diagnosis of diabetes at ≥20 years with no history of ketoacidosis.	*Training:* A counselling visit by a dietician; 12 bi-weekly group education sessions led by ‘CDAs’; lay, selected based on church employees and trained over 1-month at each church.	SM, PROMS
				*Frequency of calls:* CDA monthly calls over 1 year.	
				*Content of calls:* Providing education; motivational interviewing; goal-setting; self-management skills and active listening skills, including motivational interviewing (informational, emotional and appraisal support).	
				*Control:* Mailing of 2 pamphlets.	
**Parry 2009**[31]	Canada	RCT 2 arm.	101 Patients first time non-emergency post CABG surgery, ready for discharge.	*Peer training:* 14 ‘Peer volunteers’ with cardiac problems were trained to share surgery experiences; provided calls for 8 weeks post discharge.	SM, PROMS, HU
				Peers focused conversations on self-management and providing encouragement to attend a rehabilitation programme.	
				*Frequency of calls:* Average of 12 calls, 30 minutes in duration.	
				*Content of calls:* Sharing experiences and active listening skills including motivational interviewing (informational, emotional and appraisal support).	
				*Control:* Received preoperative and postoperative education.	
**Batik 2008**[32]	US	RCT 2 arm: Patient level	14 Patients with type 2 diabetes ≥ 65 years.	*Training:* Volunteers (number unclear); active, older adults, already engaged in senior centre programs, provided ‘lay’ motivational telephone support for 6 months. Training involved learning how to increase self-efficacy in relation to individuals’ readiness to change.	SM, HU
				*Frequency of calls:* The frequency and number of calls is unclear.	
				*Content of calls:* Increasing physical activity levels rather than heart rate goals; increasing self-efficacy and self-management skills; and listening skills, including motivational interviewing (informational, appraisal and emotional support).	
				*Control:* Delayed PALS intervention 1 year on.	
**Carroll 2007**[33]	US	RCT 2 arm: Patient level	247 Unpartnered patients post MI and CABG surgery ≥ 65 years.	*Peer training*: Practice nurses trained peers according to a validated peer training program involving elders with MI. Peer advisers were matched to patient participants in relation to age and gender.	SM, HU
				*Frequency of calls:* 1 Community based home-visitwithin 72 hours and calls at 2, 6, and 10 weeks from a nurse and 12 weekly telephone calls from 45 ‘peer advisors’.	
				*Content of calls:* Imparting cardiac information; motivational interviewing; implementing social support and increasing self-efficacy to improve physical and mental health (informational, appraisal and emotional support).	
				*Control:* Usual care.	
**Young 2005**[34]	UK	RCT 2-arm: Patient-level.	591 Patients with type 2 diabetes with diagnosis ≥ I year.	*Training:* ‘PACCTS’ delivered by lay ‘telecarers’ with support on treatment changes from DSN. DSNs delivered 3-month training program to telecarers on principles of: managing type 2 diabetes; self-management; communication skills; focussed listening; building and managing a telephone relationship; change management; motivational interviewing; and use of the PACCTS application.	HU, SM
				*Frequency of calls:* Calls performed every 3-months if HbA1c was <7%; every 7 weeks if HbA1c was in the range of 7.1-9%; and monthly if HbA1c was >9%. PACCTS application scheduled calls based on HbA1c reading.	
				*Content of calls:* Knowledge about diabetes; smoking cessation; medication adherence; motivational interviewing; and active listening skills (informational, emotional and appraisal support).	
				*Control:* Usual care including lifestyle advice and drug treatment following local guidelines including comprehensive annual review.	
**Keyserling 2002**[35]	US	*RCT 3-arm. Stratified by practice: Patient and clinician level.	200 African American women with type 2 diabetes, defined as diagnosis of diabetes at ≥20 years with no history of ketoacidosis.	*Peer training*: 4 Weekly, 4 hourly training sessions by community advisor; sessions were designed to promote readiness to change behaviours and social support.	SM, PROMS
				*Frequency of calls:* Group A: Clinic and community – 4 monthly visits with nutritionist to enhance physical activity and diet tailored to baseline attitudes. 3 group sessions and 12 monthly phone calls from ‘peer counsellor’ designed to provide social support and reinforce behaviour change; Group B: Clinic only - 3 monthly visits with nutritionist to enhance physical activity and diet tailored to baseline attitudes.	
				*Content of calls:* Promoting and maintaining healthy eating and physical activity; medication adherence; implementing self-management based on behaviour change theory and motivational interviewing (informational, emotional and appraisal support).	
				*Control:* Group C: ‘Minimal intervention’ education pamphlets mailed to participants.	

Note: Outcomes of included studies: SM = Self-management; CO = Clinical outcomes; PROMS = Patient-reported outcome measures; HU = Health utilisation; *One arm ineligible for inclusion in our anlaysis.

#### Setting

Seven studies were conducted in the United States (US: [27-30,32,33,35]); one in Canada [31] and; two in the UK [9,34]. Eight studies were based within a primary care and community setting [9,27,28,30,32-35]; one study was based in a secondary care setting [31]; and one study was based in a US Department of Veterans Affairs [29].

#### Design

Nine studies were individually randomised [9,27-35], and one cluster randomised [30]. Although two studies included two interventions against a single control [9,35], in both cases only one intervention arm met our inclusion criteria.

#### Participants and interventions

Seven studies included participants with diagnoses of diabetes [9,28-30,32,34,35], two studies included participants with heart disease (post CABG surgery [31,33]) and one study included participants with uncontrolled hypertension [27].

In seven studies, the intervention was delivered by ‘peers’ who were recruited specifically on the basis of characteristics shared with the target population [9,27,29-31,33,35]. In the other three studies, ‘lay health workers’ were recruited on the basis of their ability to communicate effectively [34], had a general willingness to help [32], or because they were judged to be ‘non clinical health educators’ [28].

The included studies used a wide variety of contexts for the recruitment of non-healthcare professionals, including recruitment from general practices [27,34]; a Department of Veteran Affairs [29]; outpatient clinics [31]; community condition-specific user groups [9]; community centres [32,35]; academic medical centres [28,33]; and recommendations from members of a church [30].

In all studies, training was based on motivational interviewing (see Additional file 2: Table S1 for additional details of the characteristics of included studies displayed in Table 1). Motivational techniques and tailoring telephone support were based on behaviour change theory, which included demonstrating active listening skills, and providing self-management support and lifestyle counselling [9,27-35].

Structured telephone support focused on increasing patient self-efficacy [9,27-30,32,33,35], and on providing social support [29,30,33,35]. Conversations involved delivery of different types of support for self-management, including: *informational support* (information on self-help services); *emotional support* (sympathy, empathy, caring) and *appraisal support* (information for self-evaluation [15,30]).

In terms of additional interventions alongside the telephone support, two studies used the telephone to additionally offer automatic call behavioural self-management reminders to the provider and patient, and prompt referral to healthcare professionals [29,34]. One study used telephone support sessions plus optional group face-to-face peer support sessions [29]. Four studies supplemented telephone support with professional counselling sessions [27,30,33,35].

### Methodological quality

Our assessments of study quality are shown in Table 2.

**Table 2:** Assessment of risk of bias of included studies

**Study**	**Random sequence (Judgment)**	**Allocation concealment (Judgment)**	**Blinding participants (Judgment)**	**Blinding outcome (Judgment)**	**Incomplete outcome (Judgment)**	**Selective reporting (Judgment)**
**Turner 2012**[27]	Randomised using a random computer sequence generation **(√)**	No information **(?)**	Attempted blinding as ALL patients received mailed brochures about heart disease.	Clinical outcomes (changes in 4 year CHD risk, systolic and diastolic blood pressure) assessors were blinded **(√)**	85% completed blood pressure assessment and 69% completed CHD risk assessment. No difference between groups. More withdrawals in intervention group (20/136 v 13/144). Multiple imputation for all missing values **(√)**	No protocol, description of clinical assessments correspond to outcomes **(X)**
			No self report outcomes **(√)**			
**Walker 2011**[28]	Randomised using a random computer sequence generation **(√)**	No information **(?)**	Attempted blinding as ALL patients received mailed brochures about heart disease. Self report outcomes used **(X)**	No blinding **(X)**	87% completed outcomes assessments at 12 months. No difference between groups. More withdrawals in control group (3/264 v 2/262). Multiple imputation for all missing values **(√)**	No protocol, description of clinical assessments correspond to outcomes **(X)**
				Outcomes self-report by telephone. Physiological measures completed using the ‘dry-dot methodology’ involving patient mailing sample to the lab **(?)**		
**Heisler 2010**[33]	Randomised using a random sequence generation **(√)**	Centrally **(√)**	Blinded patients, research staff and care managers at baseline. Intervention was described as a comparison of 2 diabetes self-management support models to participants. Not clear after baseline **(X)**	Only data assessors were blinded **(X)**	89% completed HbA1c assessments and 95% completed survey assessments, no differences between groups, justification is provided **(√)**	No protocol, description of measures orresponds to outcomes **(X)**
**Dale 2009**[9]	No details about sequence generation – states randomised only **(?)**	Opaque sealed envelopes **(X)**	Attempted blinding as ALL patients received one telephone call **(X)**	Outcomes self report by post **(?)**	91% follow up at 6 months (93.3%, 86.4% and 91.8% overall) no reasons given **(?)**	Protocol reported diabetes self care activities measure which was not reported in the main trial **(X)**
				Physiological measures assessed blinded to group **(√)**		
**Samuel-Hodge 2009**[32]	Cluster randomised. Computer generated random number **(√)**	Sequentially numbered sealed envelopes **(X)**	No blinding, self report outcome **(X)**	HbA1c measures masked to study group **(√)**	87% follow up at 8 months, 85% at 12 months, no difference between groups, more withdrawals in intervention group (6/102 v 1/72) **(√)**	No protocol, insufficient information **(?)**
				Physical activity not clear **(?)**		
				FFQ and other psychosocial outcomes by telephone, masked to study group but not clear if it could have been broken (**?)**		
**Parry 2009**[34]	Internet based randomisation service (**√)**	Central (**√)**	No blinding, self report outcome (**X)**	Researchers blinded to group allocation, self reported outcomes, but not clear if could have been broken (**?)**	Follow up 94% at 8 weeks, no difference between groups, reasons given (**√)**	No protocol, insufficient information (**?)**
**Batik 2008**[30]	Non random assignment of late new participants to control group **(X)**	No information **(?)**	No blinding, self report outcome **(X)**	Outcomes self-report **(?)**	No data reported on follow up **(?)**	No protocol, insufficient information **(?)**
				Physiological measures **(?)**		
**Carroll 2007**[31]	No details about sequence generation, states randomised only **(?)**	No information **(?)**	No blinding, self report outcome **(X)**	Outcomes self-report via telephone **(?)**	18.6% attrition, no reasons given **(?)**	No protocol, insufficient information **(?)**
**Young 2005**[35]	Post-recruitment block randomisation, stratified by baseline HbA1c using SAS software **(√)**	Randomise intervention to control in a ratio of 2:1 **(√)**	No information **(?)**	No information **(?)**	8.2% lost at follow-up, justification is provided, intention to treat analyses **(√)**	No information **(?)**
**Keyserling 2002**[29]	Randomised using random numbers generated using a personal computer **(√)**	Consequently numbered sealed envelopes containing study group assignments **(X)**	No blinding, self report outcome **(X)**	Clinicians were informed of participants group assignment, no more information is provided **(X)**	88% and 84% of participants completed the 6^th^ and 12^th^ month follow-up, no differences between groups, justification is provided **(√)**	Protocol includes self-care, but no outcomes are reported **(X)**

Note: Judgment ratings: **√** = Low risk of bias; **X** = High risk of bias; **?** = Unclear risk of bias [22].

**Figure 2:**
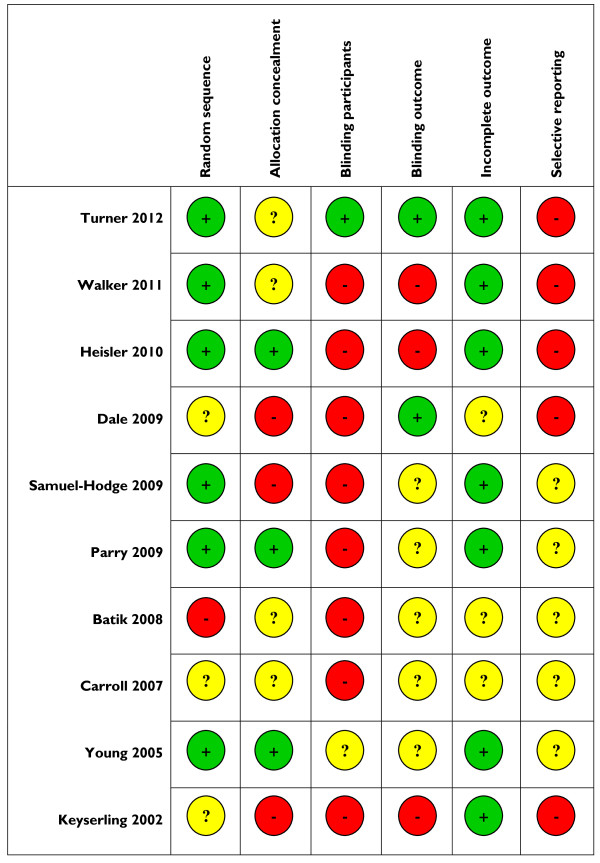
**Risk of bias summary: review authors’ judgements about each risk of bias item for each individual study, adapted from Higgins and colleagues **[22]**.**

Many included studies were at risk of bias. Overall, the most recent study by Turner and colleagues (2012) was judged to be of a better quality in terms of randomisation, blinding and incomplete outcome reporting [27]. A summary of the risk of bias in included studies is presented in Figure 2.

### Outcomes

**Figure 3:**
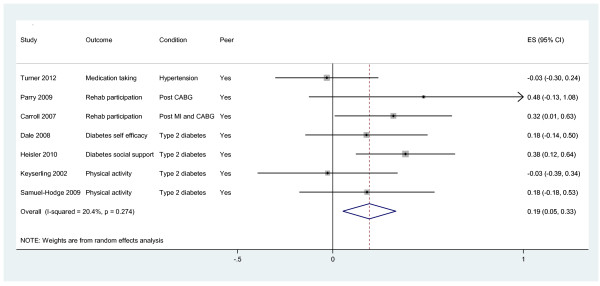
Effects of peer telephone support on self-management.

**Figure 4:**
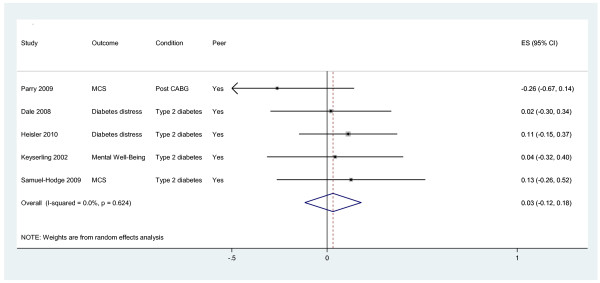
Effects of peer telephone support on mental health quality of life.

**Figure 5:**
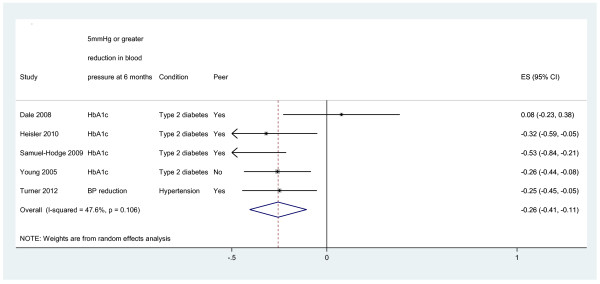
Effects of peer telephone support on clinical (surrogate outcomes).

Outcomes are presented in Figures 3, 4, 5. We present pooled the analyses for self-management, mental health quality of life and surrogate outcomes only, as in these cases there were sufficient data (i.e. more than two studies).

#### Self-management

Seven studies reported effects on self-management behaviour [9,27,29-31,33,35]. Self-management behaviours included medication taking, participation in cardiac rehabilitation programmes, physical activity, social support and self-efficacy. The pooled effect across studies was 0.19 (95% CI 0.05 to 0.33, I^2^ = 20.4%; see Figure 3). When the analyses were restricted to studies in diabetes [9,29,30,34,35], the pooled effect across studies was 0.21 (95% CI 0.04 to 0.38, I^2^ = 10.1%).

#### Mental health

Five studies reported effects on mental health and distress outcomes [9,29-31,35]. Outcomes included scores on the mental component of the SF-36 (MCS: [30,31]) and scores on diabetes problem areas [9] and distress questionnaires [29,35]. The pooled effect across studies was 0.03 (95% CI −0.12 to 0.18, I^2^ = 0%; see Figure 4). When the analyses were restricted to studies in diabetes [9,29,30,34,35], the pooled effect across studies was 0.08 (95% CI −0.08 to 0.24, I^2^ = 0%).

#### Clinical (surrogate outcomes)

Four studies reported effects on HbA1c [9,29,30,34]. The pooled effect across studies was -0.26 (95% CI -0.41 to -0.11, I^2^ = 47.6%; see Figure 5). One study reported effects on blood pressure (−0.25, 95% CI −0.45 to −0.05; [27]).

#### Health care utilisation, costs and cost effectiveness

Only two studies reported analysable data on health care utilization: acute hospitalizations at 6 months and visits to a dietician [33,35]. In both studies, negative effect sizes indicate lower health care utilization in the peer telephone support group compared to control group. Carroll and colleagues reported a large but non-significant effect on acute hospitalizations at 6 months (SMD = −0.84, 95% CI = −1.69 to 0.02; [33]). Keyserling and colleagues reported a moderate but non-significant effect on dietician visits (SMD = −0.33, 95% CI = −0.75 to 0.10; [35]).

No studies reported costs or cost effectiveness analyses.

## Discussion

### Principal findings

This review identified ten RCTs that were designed to assess the effectiveness of telephone self-management support by ‘lay health workers’ and ‘peer support workers’ for the prevention and management of vascular disease. The studies reviewed were: primarily based in community settings in the USA; with participants who had diabetes; and used ‘peers’ recruited on the basis that they shared characteristics with patients.

The pooled analyses showed evidence of modest effects on self-management and HbA1c, but no effect on mental health quality of life. None of the studies reported data on cost effectiveness and the data on health care utilisation was very limited. The limited evidence base available to the review meant that we were unable to investigate differences in effectiveness between ‘lay health workers and ‘peer support workers’, or relationships with type or intensity of self-management support, or with study quality.

### Comparisons with other reviews

Our focus on ‘lay health workers’ and ‘peer support workers’ and vascular disease and related disorders was novel, as other published reviews on this subject have focused on the assessment of healthcare based professional support and have been disease specific [14], or reported the effects of peer delivered telephone support, on a far wider range of disorders and health problems [13].

Building on the latter review [13], we showed how non-healthcare professionals have the potential to show modest effects on HbA1c in patients. Similar to the other review, we also found no evidence relating to the cost-effectiveness of such interventions; thus we could not determine whether if such interventions are potentially low cost alternatives to professionally delivered care [13].

### Strengths and weaknesses

We utilised an efficient search strategy which does not involve multiple databases, but which takes advantage of the comprehensive nature of the CENTRAL database and has been proven to be an effective search strategy. We also complemented this search by checking of published reviews [13,14,36-41].

Given the small number of included studies, there is an argument that our inclusion criteria were too narrow, for example excluding ‘telemedicine’ studies. However, our criteria was based on previous reviews which distinguish between ‘telemedicine’ and ‘telephone support’ [14]. Further, in our searches we found no telemedicine interventions delivered by non-healthcare professionals.

The only available clinical outcome for diabetes control was a surrogate outcome involving HbA1c level, and we found no data on outcomes such as CVD, disability, or mortality [42].

Many of the studies were at potential risk of bias, as many studies were rated as ‘unclear’ because of a lack of information. We were limited to the published information and the failure to publish detail or study protocols made assessments (especially of selective outcome reporting) difficult. Unfortunately we also had inadequate review resources to clarify our judgement ratings by contacting authors.

The small number of studies makes visual or statistical assessment of publication bias through a funnel plot problematic. However, caution is warranted in the context of a number of small studies predominately showing beneficial effects.

Approximately 40% of patients with diabetes have associated CKD [43]. Although our aim was to support the development of an intervention in CKD, we thought that practically, there were unlikely to be enough studies to support a CKD-specific review. We have assumed enough commonalities in the management of vascular disorder to make the results potentially generalisable across this cluster of disorders. However, there remains limited understanding of optimal self-management of CKD [44]. Whilst the maintenance of vascular health and prevention of progression of kidney disease are central to the management of people with CKD [4,18], there is increasing evidence that care for people with early stage CKD should also focus on the prevention and management of acute kidney injury (AKI: [45]). AKI is common and preventable through better management of episodes of acute illness (e.g. sepsis due to flu), particularly in the elderly and those with multimorbidity, the prevalence of which is greater in areas of socioeconomic deprivation [44,46,47]. Although AKI is associated with poor health outcomes and increased utilisation of healthcare resources and prevention centres, currently there is a limited evidence base concerning its prevention in the community [46,47].

## Conclusions

Our findings reinforce evidence from published reviews of peer delivered support that suggest benefits on certain self-management and clinical outcomes, but limited evidence of impact on other outcomes, such as quality of life, health utilisation and cost-effectiveness [13,36]. The review findings are limited by the small number and heterogeneity of studies.

Overall, we highlight a need for well designed trials assessing ‘lay health worker’ and ‘peer support worker’ telephone support for the prevention and management of vascular disease, to identify the effective components on health outcomes, and to assess cost effectiveness. At present, there is insufficient data in the quantitative literature to fully inform the development of such interventions. Until such data are available, development will be dependent on theoretical considerations, findings from patient experience studies, and indirect evidence from other data of effective components in the wider self-management literature.

## Abbreviations

CVD: Cardiovascular disease; CKD: Chronic kidney disease; AKI: Acute kidney injury; BP: Blood pressure; HbA1c: Glycated hemoglobin; CDSR: Cochrane Database of Systematic Reviews; DARE: Database of Abstracts of Reviews of Effects; CENTRAL: Cochrane Central Register of Controlled Trials; RCTs: Randomised controlled trials; CONSORT: Consolidated Standards of Reporting Trials; GPs: General practitioners; SMD: Standardised mean difference; SF-36: 36-item short form health survey; MCS: Mental component of the SF-36; SM: Self-management; CO: Clinical outcomes; PROMS: Patient-reported outcome measures; HU: Health utilisation; CDA: Church Diabetes Advisors; PACCTS: Pro-Active Call Centre Treatment Support; DSN: Diabetes specialist nurse; US: United States; UK: United Kingdom; AHA: American Heart Association; CLAHRC: Collaboration for Leadership in Applied Health Research and Care; BRIGHT: BRinging Information and Guided Help Together; NIHR: National Institute of Health Research; NICE: National Institute for Health and Care Excellence; FSF: Flexibility and sustainability funding

## Competing interests

The authors declare that they have no competing interests.

## Authors’ contributions

NS, PB, TB and CCG developed the idea for the study. NS devised the search strategy, reviewed articles, extracted the data and interpreted the results. MP and PB conducted the analysis. PB, MP and CB assisted with the data extraction. NS drafted the manuscript. All authors read and approved the final manuscript.

## Authors’ information

^1^NS is a Research Associate at the Centre for Primary Care and was a collaborator on the Greater Manchester CLAHRC, Patient Theme. Her research is mainly within the Long-Term Conditions theme, focusing on patient health and illness experience.

^1^CB is a Research Associate at the Centre for Primary Care within the Long-Term Conditions theme.

^1^TB is a General Practitioner (GP) and NIHR Clinical Lecturer at the Centre for Primary Care.

Both CB and TB are joint Principal Investigators on the BRIGHT (BRinging Information and Guided Help Together) RCT which is currently evaluating a complex self-management intervention aimed at improving outcomes for patients with stage 3 CKD. This RCT forms a key element of the CLAHRC for Greater Manchester, which aims to improve the quality of vascular care for people with vascular diseases.

^1^MP is a Research Associate at the Centre for Primary Care and was a collaborator on the Greater Manchester CLAHRC, Patient Theme.

^2^CCG is a GP and Professor of GP Research at Keele University and has an Honorary Professorship at the University of Manchester.

^1^PB leads the Centre for Primary Care in the Institute of Population Health, which is part of the NIHR School for Primary Care Research and also works within the CLAHRC for Greater Manchester, Patient and Practitioner Theme. His work is largely within the Long-Term Conditions theme, with a focus on mental health, multimorbidity, and service delivery.

## Pre-publication history

The pre-publication history for this paper can be accessed here:

http://www.biomedcentral.com/1472-6963/13/533/prepub

## Supplementary Material

Additional file 1 Full search strategy. Click here for file

Additional file 2: Table S1 Additional characteristics of included studies. Click here for file
