# Supplementary material for: Telephone based self-management support by ‘lay health workers’ and ‘peer support workers’ to prevent and manage vascular diseases: a systematic review and meta-analysis
Source: BMC Health Serv Res. 2013 Dec 27;13:533. doi: 10.1186/1472-6963-13-533 (PMC3880982; doi:10.1186/1472-6963-13-533)
Supplement: Additional file 1 — Full search strategy. [file 1472-6963-13-533-S1.pdf]

Additional file 1 Full search strategy

Database: EBM Reviews - Cochrane Central Register of Controlled Trials <March 2013>
Search Strategy:
----
1 exp vascular diseases/ (47049)
2 vascular.tw. (12203)
3 exp Kidney Failure, Chronic/ (2735)
4 chronic kidney disease$.tw. (854)
5 exp cardiovascular diseases/ (59831)
6 cardiovascular.tw. (16915)
7 exp cerebrovascular disorders/ (6749)
8 cerebrovascular.tw. (1803)
9 exp peripheral vascular diseases/ (1973)
10 (peripheral adj5 disease).tw. (1520)
11 exp Myocardial Isch?emia/ (17949)
12 (isch?emic adj5 disease).tw. (1620)
13 exp stroke/ (3311)
14 exp isch?emic Attack, Transient/ (423)
15 isch$emic.tw. (6990)
16 exp heart failure/ (4376)
17 heart failure.tw. (7614)
18 cardiac failure.tw. (420)
19 exp diabetes mellitus/ (12444)
20 diab$.tw. (21881)
21 exp hypertension/ (12373)
22 hypertensi$.tw. (22979)
23 or/1-22 (107364)
24 exp telecommunications/ (2278)
25 (telemetr$ or telemed$ or tele?med$ or telehealth$ or tele?health$ or telecare or tele?care or telehome or tele?home).tw. (580)
26 (telemonitor$ or tele?monit$ or teleconsult$ or tele?consult$ or teleconferenc$ or telecommunicat$ or tele?communicat$).tw. (255)
27 (telephon$ or phones$).tw. (4418)
28 exp remote consultation/ (200)
29 (remote$ adj (consult$ or monitor$)).tw. (36)
30 or/24-29 (5893)
31 23 and 30 (1172)

Database: Embase <1980 to 2013 Week 15>
Search Strategy:
----
1 exp vascular diseases/ (1627485)
2 vascular.tw. (488859)
3 exp Kidney Failure, Chronic/ (53353)
4 chronic kidney disease$.tw. (22115)
5 exp cardiovascular diseases/ (2697417)
6 cardiovascular.tw. (334125)
7 exp cerebrovascular disorders/ (328828)
8 cerebrovascular.tw. (45217)
9 exp peripheral vascular diseases/ (1144749)
10 (peripheral adj5 disease).tw. (26264)
11 exp Myocardial Isch?emia/ (68127)
12 (isch?emic adj5 disease).tw. (39358)
13 exp stroke/ (50733)
14 exp isch?emic Attack, Transient/ (22504)
15 isch$emic.tw. (175999)
16 exp heart failure/ (259546)
17 heart failure.tw. (136667)
18 cardiac failure.tw. (12063)
19 exp diabetes mellitus/ (531666)
20 diab$.tw. (503033)
21 exp hypertension/ (434720)
22 hypertensi$.tw. (387633)
23 or/1-22 (3522640)
24 exp telecommunications/ (32452)
25 (telemetr$ or telemed$ or tele?med$ or telehealth$ or tele?health$ or telecare or tele?care or telehome or tele?home).tw. (15540)
26 (telemonitor$ or tele?monit$ or teleconsult$ or tele?consult$ or teleconferenc$ or telecommunicat$ or tele?communicat$).tw. (4700)
27 (telephon$ or phones$).tw. (49819)
28 exp remote consultation/ (5017)
29 (remote$ adj (consult$ or monitor$)).tw. (1212)
30 or/24-29 (87541)
31 random$.af. (920049)
32 23 and 30 and 31 (2621)

Database: Ovid MEDLINE(R) <1946 to April Week 2 2013>
Search Strategy:
----
1 exp vascular diseases/ (1261988)
2 vascular.tw. (378020)
3 exp Kidney Failure, Chronic/ (74607)
4 chronic kidney disease$.tw. (14279)
5 exp cardiovascular diseases/ (1774871)
6 cardiovascular.tw. (235559)
7 exp cerebrovascular disorders/ (259755)
8 cerebrovascular.tw. (33423)
9 exp peripheral vascular diseases/ (42327)
10 (peripheral adj5 disease).tw. (18534)
11 exp Myocardial Isch?emia/ (339876)
12 (isch?emic adj5 disease).tw. (30158)
13 exp stroke/ (76211)
14 exp isch?emic Attack, Transient/ (16996)
15 isch$emic.tw. (127710)
16 exp heart failure/ (80812)
17 heart failure.tw. (92579)
18 cardiac failure.tw. (9300)
19 exp diabetes mellitus/ (294064)
20 diab$.tw. (357049)
21 exp hypertension/ (200822)
22 hypertensi$.tw. (284941)
23 or/1-22 (2519736)
24 exp telecommunications/ (56255)
25 (telemetr$ or telemed$ or tele?med$ or telehealth$ or tele?health$ or telecare or tele?care or telehome or tele?home).tw. (11995)
26 (telemonitor$ or tele?monit$ or teleconsult$ or tele?consult$ or teleconferenc$ or telecommunicat$ or tele?communicat$).tw. (3582)
27 (telephon$ or phones$).tw. (38145)
28 exp remote consultation/ (3375)
29 (remote$ adj (consult$ or monitor$)).tw. (687)
30 or/24-29 (90062)
31 random$.af. (767789)
32 23 and 30 and 31 (1987)
