# Supplementary material for: Telephone based self-management support by ‘lay health workers’ and ‘peer support workers’ to prevent and manage vascular diseases: a systematic review and meta-analysis
Source: BMC Health Serv Res. 2013 Dec 27;13:533. doi: 10.1186/1472-6963-13-533 (PMC3880982; doi:10.1186/1472-6963-13-533)
Supplement: Additional file 2: Table S1 — Additional characteristics of included studies. [file 1472-6963-13-533-S2.pdf]

Additional file 2 Table S1. Additional characteristics of included studies

Turner 2012 [26]

**Methods**	*Setting:* Two adjacent urban academic primary care practices with a low-income minority patient population in the US.<br>*Recruitment:* Identified 9135 African-American patients from patient records aged 40 – 75 years, with over two practice visits in 2 consecutive years.<br>*Randomisation:* RCT 2 arm.<br>*Definition of non-health care professionals:* 'Peer-patients' (coaches with controlled hypertension) were identified from patient records by health care professionals who were: aged 50 – 75 years; had well-controlled hypertension; were good communicators; compliant to medical care and were judged to be 'empowered' to manage their condition.<br>*Peer training:* Peer-patients were taught motivational interviewing skills from an experienced lead peer coach during two and a half face-to-face training sessions. Training content was developed by an African-American community advisory board to address barriers and facilitators to CHD risk reduction.
**Participants**	280 African-American patients with uncontrolled hypertension.
**Interventions**	*Intervention:* Three monthly calls from 11 trained peer-patients plus two practice staff visits (involving 3 members of staff; a medical assistant; practice nurse; and chronic disease health educator) to review a personalised 4-year heart disease risk calculator and view slide shows about heart disease risks. Peer callers addressed attitudes in line with the Theory of Planned Behaviour which involved using role modelling techniques and offering evidence-based advice.<br>*Caller:* Peer-patients (coaches).<br>*Control group:* Usual physician care and written healthy cooking advice via heart disease brochures.
**Outcomes**	*Self management:* None recorded.<br>*PROMS:* None recorded.<br>*Clinical outcomes:* 4-year CHD risk measure to assess risk of a primary or secondary CHD event; change in HbA1c level.<br>*Health utilisation:* None recorded.<br>*Costs:* None recorded.

Additional file 2 Table S1. Additional characteristics of included studies

Walker 2011 [27]

**Methods**	*Setting:* Einstein Diabetes Research and Training Centre with access to a low income, insured (members of a union with a jointly sponsored health benefit plan), minority patient population in New York.<br>*Recruitment:* Eligible patient participants identified through a database were telephoned and completed a screening measure. Potential participants were mailed an HBA1c blood test kit and enrolled if their result was ≥7.5% then randomised to either group.<br>*Randomisation:* RCT 2 arm.<br>*Definition of non-health care professionals:* No definition; non clinical 'health educators' were used (no description of recruitment or numbers of health educators reported).<br>*Lay training:* Trained and supervised by diabetes educator nurse.
**Participants**	526 Adult (≥ 30 years of age) members of the health care worker union fund in New York; read and spoke English or Spanish with no evidence of cognitive impairment; diabetes prescription of at least one oral glucose lowering agent in the year prior to enrolment; eligible A1c was 7.5% to provide a margin for lowering the HbA1c.
**Interventions**	Monthly calls for a year from health educator (length not reported). Calls were tailored to each patient and focused on diabetes medication adherence, making behavioural lifestyle changes through eating healthily and exercising. Telephone support manuals were used to guide conversations based on self-efficacy and empowerment and were informed by the Theory of Planned Behaviour. Also received high quality self-management printed materials by mail and prompted during telephone conversations to use these materials.<br>*Caller:* Health educators.<br>*Control group:* Self-management printed materials by mail.
**Outcomes**	*Self management/ PROMS:* Self-reported medication-taking; Self care medication adherence (medication possession ratio)<br>*Clinical outcomes:* Change in HbA1c level.<br>*Health utilisation:* None recorded.<br>*Costs:* None recorded.

Additional file 2 Table S1. Additional characteristics of included studies

Heisler 2010 [32]

**Methods**	*Setting:* 2 Midwestern U.S. Department of Veterans Affairs.<br>*Recruitment:* Recruited cohorts 45-66 years+ to facilitate group sessions and pair patients with an age-matched peer partner.<br>*Randomisation:* RCT 2 arm<br>*Definition of non-health care professionals:* 'Allows patients' to share experiences and receive reinforcement that is not available from time-pressed clinicians, and it may especially benefit patients who are tackling challenging medical tasks, such as insulin management'.<br>*Peer training:* Peers attended a group session to set diabetes goals, receive peer communication skills training, and receive peer support from an age-matched 'peer partner'.
**Participants**	244 Patients with diabetes; Peers (n = 125); Nurse care management (n = 119).
**Interventions**	Peers were encouraged to talk weekly using a telephone that recorded call occurrence and provided reminders to initiate peer contact. Optional group peer-led sessions at 1, 3, and 6 months were available.<br>*Caller:* Peer partners.<br>*Control group:* Enhanced usual care consisting of an educational session and an assigned nurse care manager.
**Outcomes**	*Mental health:* Diabetes distress.<br>*Self management/ PROMS***:** Diabetes-specific social support, medication adherence.<br>*Clinical outcomes:* HbA1c level.<br>*Health utilisation:* Reviewed medical records to determine number of primary care and diabetes clinic visits.<br>*Costs:* None recorded.

Additional file 2 Table S1. Additional characteristics of included studies

Dale 2009 [9]

**Methods**	*Setting:* 43 General practices Warwickshire, Coventry, UK.<br>*Recruitment:* Potential patient participants recruited from 3 general practice clinics; Diabetes Specialist Nurses (DSNs) recruited through DSN directory; Peers recruited through Warwick Diabetes Care User Group, plus email support group. Asked to give their experience on offering telephone advice, counselling and reasons for participating in the study. Engaged and interested participants were allocated roles and paid a small amount (not reported).<br>*Randomisation:* RCT 3 arm<br>*Definition of non-health care professionals:* 'Based on the concept of sharing mutual experience and experiential knowledge' benefiting the peer and the participant by increasing feelings of self worth and changes in self-management behaviour'.<br>*Peer training:* Peers attended a two day training programme developed for the study which focused on empowerment, motivational interviewing, active listening skills, and telephone role playing.
**Participants**	231 Patients with diabetes; 9 Peers (males n = 4; females n = 5; age range 35 – 75 years; type 2 diabetes n = 6; 5 – 28 years duration of diabetes);12 DSNs (all female; 35 – 63 years age range; 6 – 22 years diabetes nursing experience; type 2 diabetes n = 1).
**Interventions**	Calls for up to 6 months. The first call was made 3-5 days later and at the following days: 7-10, 14-18, 28-35, 56-70, 120-150. More intense reinforcement of behaviour change occurred during the early weeks following initiation. The frequency of calls was intended to be tailored to patients' individual needs and callers were taught to negotiate the time of subsequent contact as part of the closure of each call.<br>*Caller:* Made telephone calls from a confidential space in the workplace or home. Invited to share challenging cases at 6 month review meetings.<br>*Control group:* Patients were informed that they were allocated to the routine care group; Received a single call from a researcher at day 3 – 5; Encouraged to follow the advice of GP or practice nurse.
**Outcomes**	*Mental health:* Diabetes distress.<br>*Self-management/ PROMS:* Diabetes self care activities; Adherence to treatment; Diabetes Management Self-Efficacy; Perceived therapeutic efficacy.<br>*Clinical outcomes:* HbA1c level.<br>*Health Utilisation:* None recorded.<br>*Cost effectiveness:* None recorded.

Additional file 2 Table S1. Additional characteristics of included studies

Samuel-Hodge et al 2009 [31]

**Methods**	*Setting:* 24 African American churches in central North Carolina.<br>*Recruitment:* 300 Churches identified from: an existing project database used in another study; community contacts and Chamber of Commerce resources. 118 Churches invited to participate.<br>8 – 20 Patient participants per church were identified by a church liaison who initiated recruitment through posters, pamphlets, and church announcements. Interested participants: called a free phone number at the research office; or sent an opt-in card to the research office; or spoke to a church liaison officer.<br>*Randomisation:* Cluster RCT 2 arm<br>*Definition of non-health care professionals:* Peer counselling was given via a 'Church Diabetes Advisor' (CDA) with type 2 diabetes or having lived with someone diagnosed with diabetes for at least 2 years.<br>*Training:* CDAs were selected based on recommendations of the church and trained over a 1-month period – 4 weekly, 4 hour sessions – in the areas of motivational interviewing techniques, listening skills, diabetes self-management, and telephone counselling.
**Participants**	20 years or older; diagnosis of type 2 diabetes, clinical care provided by a primary care practitioner; plans to reside within 50 miles of church for 1 year; and telephone access.
**Interventions**	Special Intervention (SI) involved 1 individual 60 minute counselling visit to a dietician to facilitate subsequent counselling by the CDAs and 12 bi-weekly group education sessions at each church (led by the dietician and assistance of a CDA), each lasting 90 – 120 minutes, encouraging behaviour change and education. CDA delivered monthly telephone calls for 1 year to offer support for behaviour change to improve diabetes self-management.<br>*Caller:* Calls made by CDAs.<br>*Control group:* Minimal Intervention (MI) was a direct mailing of 2 pamphlets ("Healthy Eating" and "Staying Active") and 3 bimonthly newsletters, published by the American Diabetes Association, providing general health information and study updates.
**Outcomes**	*Mental health:* General health.<br>*Self management/ PROMS:* Amount of physical activity; Diabetes related knowledge; Diabetes-related health status; dietary intake.<br>*Clinical outcomes:* Change in HbA1c level (determined by a finger sample collected at participant's church).<br>*Health utilisation:* None recorded.<br>*Costs:* None recorded.

Additional file 2 Table S1. Additional characteristics of included studies

Parry 2009 [33]

**Methods**	*Setting:* Single clinic in Toronto, Ontario, Canada.<br>*Recruitment:* Peer volunteers were recruited during February and March 2006 via letters, advertisements in local newspapers and posters displayed at the local outpatient cardiac rehabilitation program. Peer volunteers were screened for their ability to engage in conversation; give information clearly; share experiences and display appropriate listening skills.<br>*Randomisation:* RCT 2 arm.<br>*Definition of non-health care professionals:* 'Peer volunteers have similar characteristics and possess specific knowledge that is concrete, pragmatic and derived from shared experiences'.<br>*Peer training:* A 4 h training session to clarify and review content materials, develop skills required for effective telephone support; to understand when and how to facilitate appropriate referrals to health professionals; and demonstrate learning through role-playing. Support was initiated within 72 h of hospital discharge and support continued for a period of eight weeks. Peer volunteers also received a training manual intended to guide the training sessions and the intervention.
**Participants**	101 patients who had undergone CABG surgery; 14 peer providers, 11 were men, married, and all were retired.
**Interventions**	Provided calls for 8 weeks following hospital discharge. Peers used the usual care materials to focus their telephone conversations on pain management, exercise and motivation to attend a cardiac rehabilitation programme.<br>*Caller:* Peer volunteers.<br>*Control group:* Patients allocated to usual care received preoperative and postoperative education, and visits from in-hospital peer volunteers.
**Outcomes**	*Mental health:* Health related quality of life.<br>*Self-management/ PROMS:* Pain and pain related interference with activities, and cardiac rehabilitation participation.<br>*Clinical outcomes:* None recorded.<br>*Health utilisation:* Feasibility measures including the Peer activity log, the Peer Recruitment and Training Evaluation Survey, and the Peer Support Evaluation Inventory.<br>*Costs:* None recorded.

Additional file 2 Table S1. Additional characteristics of included studies

Batik 2008 [29]

**Methods**	*Setting:* Two community clinics in the Southeast Seattle; the neighbourhood senior centre; a community social services provider; and a Health Promotion Research Centre.<br>*Recruitment:* Staff of the neighbourhood senior centre recruited 'lay' telephone volunteers, from 'active older adults' already engaged in senior centre programs. Primary skills sought in a lay coordinator were: an ability to communicate effectively; a genuine interest in working with older adults; experience in engaging and motivating volunteers; and a personal commitment to being physically active. Primary care providers approached potential participants during consultations and measured their physical activity level. Patients who expressed interest signed a consent form permitting the sharing of their name, contact information, and exercise prescription with community partners. A referral was then faxed to the PALS project coordinator, who arranged an intake interview.<br>*Randomisation:* RCT 2 arm<br>*Definition of non-health care professionals:* Lay support was offered by training older volunteers to provide telephone support and was based on a behavioural self-efficacy programme.<br>*Training:* Training for telephone volunteers was conducted by Active Choices staff who consulted a validated peer support programme - the Physical Activity Intervention (PALS).
**Participants**	14 Patients with diabetes aged 65 years or older who had visited a clinic within the previous 18 months and who had telephone access.
**Interventions**	Volunteers provided ongoing telephone support for 6 months. The frequency and number of calls is unclear. The content of calls involved focusing on increasing physical activity levels rather than on heart rate goals.<br>*Caller:* Calls made by lay adult volunteers.<br>*Control group:* Delayed PALS intervention 1 year on.
**Outcomes**	*Self-management:* Level of physical activity (No useable data reported). *PROMS:* None recorded.<br>*Clinical Outcomes:* None recorded.<br>*Health Utilisation:* Number of follow-up clinic visits (No useable data reported)<br>*Cost effectiveness:* None recorded.

Additional file 2 Table S1. Additional characteristics of included studies

Carroll 2007 [30]

**Methods**	*Setting:* East and West coast of United States; 5 Academic medical centres.<br>*Recruitment:* All participants recruited from cardiac rehabilitation programs; Peer advisors were older than 60 years, with a history of MI and CABS, had a successful completion of a cardiac rehabilitation program, and were actively participating in a healthy lifestyle.<br>*Randomisation:* RCT 2 arm.<br>*Definition of non-health care professionals:* Collaborative Peer Advisor and Advanced Practice Nurse Intervention based on self-efficacy and social support enhancement to improve the physical and mental health of participants.<br>*Peer training:* Standardised training for peer advisors and close contact with practice nurses according to a validated peer training program involving elders with MI. Peer advisers were matched to patient participants in relation to age and gender.
**Participants**	247 Unpartnered (single, widowed divorced) adults; older than 65 years; post MI; able to speak English; and had access to a telephone. 45 Peer advisors trained; 2 advanced practice nurses were masters level students in cardiovascular nursing.
**Interventions**	Community based; home-visit within 72 hours; and telephone calls at 2, 6, and 10 weeks from an advanced practice nurse; and 12 weekly telephone calls from a peer advisor.<br>*Caller:* Calls made by advanced practice nurse or peer advisor.<br>*Control group:* Usual care.
**Outcomes**	*Self management:* Participation in cardiac rehabilitation.<br>*PROMS:* None recorded (although mental health was described to be assessed in the introduction).<br>*Clinical outcomes:* None recorded.<br>*Health utilisation:* Participation in cardiac rehabilitation program and re-hospitalizations reported (based on patient self report).<br>*Costs:* None recorded.

Additional file 2 Table S1. Additional characteristics of included studies

Young 2005 [34]

**Methods**	*Setting:* 25 General practices, Salford, UK.<br>*Recruitment:* Call centre staff ('lay telecarers') recruited from the general public (with excellent telephone manner) who had: experience of working with the general public; word processing skills; and able to work early and late shifts.<br>*Randomisation:* RCT 2-arm.<br>*Definition of non-health care professionals:* Previously untrained 'lay telecarers' supported by a diabetes specialist nurse to help to improve glucose control by promoting lifestyle management.<br>*Training:* 3 month training program comprising of: principles of managing diabetes; telephone motivational interviewing, including identifying health beliefs using the call centre technology.
**Participants**	591 Patients with diabetes; Specialist diabetes nurse; 2 Telecarers.
**Interventions**	Pro-Active Call Centre Treatment Support (PACCTS) interventions were outbound calls using call centre telephones (Cisco Systems equipment) and trained call centre staff to 'support and guide the patient as an individual toward achieving the best possible management of their diabetes'.<br>*Caller:* 'Lay telecarers'.<br>*Control group:* Lifestyle advice and drug treatment following local guidelines, including comprehensive annual review.
**Outcomes**	*Self management:* None recorded.<br>*PROMS:* None recorded.<br>*Clinical outcomes:* HbA1c levels.<br>*Health utilisation:* Referrals from the telecarers, telephone consultations.<br>*Costs:* None recorded.

Additional file 2 Table S1. Additional characteristics of included studies

Keyserling 2002 [28]

**Methods**	*Setting:* General practices, including 5 community health centres, a staff health maintenance organisation, and a general medicine clinic at an academic health centre, central North Carolina.<br>*Recruitment:* Nutritionists and peer counsellors.<br>*Randomisation:* RCT 3-arm.<br>*Definition of non-health care professionals:* 'Peer counsellors'.<br>*Peer training:* 4 Weekly, hourly sessions designed to promote readiness to change and diet behaviours and social support.
**Participants**	200 African American, older, lower income women with diabetes.
**Interventions**	*Intervention A:* Community and clinic component = '*A New Leaf… Choices for Healthy Living with Diabetes*' was based on behaviour change theory, promoting lifestyle and leisure-time activities, and consisting of 12 monthly telephone counselling calls and 1 group counselling session.<br>*Caller:* Peer counsellors.<br>*Intervention B:* Clinic only = 4 monthly visits with nutritionist to enhance physical activity and diet.<br>*Control group:* Education pamphlets mailed to participants.
**Outcomes**	*Mental health:* Mental well-being.<br>*Self management:* Physical activity; diet control of blood glucose.<br>*PROMS:* Diabetes knowledge; diabetes specific health status; program acceptability.<br>*Clinical outcomes:* None recorded.<br>*Health utilisation:* None recorded.<br>*Costs:* None recorded.
